# Cardiac Autonomic Measures Predict Clinician-Rated Anxiety and Behavioral Response to Propranolol in Autistic Children and Young Adults

**DOI:** 10.3390/jpm15070286

**Published:** 2025-07-03

**Authors:** Carrina Appling, Nanan Nuraini, Ryan Holem, Samantha Hunter, Kathy Hirst, Nicole Takahashi, Micah O. Mazurek, Stephen M. Kanne, Bradley Ferguson, David Q. Beversdorf

**Affiliations:** 1 Interdisciplinary Neuroscience Program, University of Missouri, Columbia, MO 65212, USA; cbaqpd@missouri.edu (C.A.); nn3w4@missouri.edu (N.N.); 2 Molecular Life Sciences Fellowship, University of Missouri, Columbia, MO 65212, USA; 3 Fulbright Fellow, University of Missouri; Columbia, MO 65212, USA; 4 School of Medicine, University of Missouri, Columbia, MO 65212, USA; rhdw4@health.missouri.edu; 5 Thompson Center for Autism & Neurodevelopment, University of Missouri Columbia, MO 65201, USAkasiahirst@gmail.com (K.H.); takahashin@health.missouri.edu (N.T.); 6 Department of Human Services, University of Virginia, Charlottesville, VA 22903, USA; mm5gt@virginia.edu; 7 Department of Health Psychology, University of Missouri, Columbia, MO 65203, USA; kannest@health.missouri.edu; 8 Department of Neurology, School of Medicine, University of Missouri, Columbia, MO 65212, USA; 9 Departments of Radiology, Neurology & Psychological Sciences, University of Missouri, Columbia, MO 65212, USA

**Keywords:** adrenergic, noradrenergic, anxiety, autism, clinical trial, heart rate variability, precision medicine, propranolol

## Abstract

Propranolol, a nonselective beta-adrenergic antagonist, has shown potential for improving anxiety in autistic individuals. Heart rate variability (HRV), a noninvasive cardiac marker of autonomic nervous system functioning, may help identify individuals most likely to benefit from propranolol. **Objectives**: Determine if baseline resting HRV and other cardiac measures predict the response to propranolol for anxiety and core autism symptomology in autistic children and young adults. **Methods:** Sixty-two autistic individuals (ages 7–24) participated in a two-phase (i.e., a 12-week randomized controlled trial and a 12-week open-label extension) trial of propranolol. Baseline (i.e., resting state, prior to treatment) HRV and other cardiac measures were obtained from an electrocardiogram. Clinical global impression for anxiety symptoms and overall behavioral treatment impact were assessed after the 12-week trial period. Group-level (i.e., all participants) and responder groups (i.e., strong, minimal, and non-responders to propranolol) were analyzed for treatment effects. **Results:** HRV variables predicted group-level anxiety response to propranolol, particularly for strong responders. Also, lower baseline values of parasympathetic HRV indices were significantly correlated with greater behavioral improvement after treatment with propranolol. Last, several baseline cardiac variables were associated with improvement in multiple behavioral domains after treatment with propranolol. **Conclusions:** HRV may be a potential biomarker for predicting reduced anxiety and behavioral symptoms in response to propranolol in autistic children and young adults. Identifying autonomic profiles associated with positive treatment outcomes could guide future personalized interventions in autism. The results presented herein should be regarded as preliminary until the findings are replicated in future clinical trials.

## 1. Introduction

Autism spectrum disorder (ASD) is conceptualized as a neurodevelopmental disorder marked by persistent deficits in social communication and social interaction across multiple contexts, coupled with restricted, repetitive patterns of behavior, interests, or activities [1]. These symptoms must be present from early developmental stages and result in clinically significant impairment in social, occupational, or other critical areas of functioning [2]. The exact cause or causes of autism are not currently known, though it is currently believed to result from an interaction between genetic and environmental factors [3,4]. Despite the high prevalence and clinical impact of ASD, pharmacological interventions directly targeting core symptoms remain limited [5,6]. Most medications prescribed to individuals with ASD are aimed at managing comorbidities, such as anxiety, irritability, or hyperactivity, but fail to address social communication or repetitive behaviors [7,8].

Propranolol, a central and peripheral nonselective beta-adrenergic antagonist, has demonstrated promise in improving social reciprocity [9] in double-blind single-dose psychopharmacological challenge studies in autistic people, as well as efficacy in reducing anxiety symptoms [10] in a double-blind, placebo-controlled trial. Propranolol is a nonselective β-adrenergic receptor antagonist that blocks both β_1_ and β_2_ receptors, thereby attenuating the physiological effects of endogenous catecholamines such as norepinephrine and epinephrine. Through β_1_-receptor antagonism, propranolol decreases cardiac output and oxygen demand, while β_2_-receptor blockade influences vascular and bronchial smooth muscle tone. Additionally, propranolol crosses the blood–brain barrier to act centrally, which may explain its therapeutic effects on anxiety and cognition [11]. As such, propranolol represents a candidate pharmacologic intervention for anxiety-related features in ASD, which are often resistant to first-line pharmacological treatments [10,12]. In addition to its anxiolytic and autonomic effects, propranolol is associated with improvements in verbal problem solving, semantic processing, working memory, and functional connectivity in ASD, suggesting broader potential for enhancing neural integration and social–cognitive processing [13].

Propranolol may also influence autonomic nervous system functioning, which is frequently altered in autistic people [14]. For example, research generally shows diminished parasympathetic tone and elevated sympathetic dominance, with high variability in autism [15,16]. Furthermore, research has shown that anxiety symptoms may help distinguish subgroups with unique physiological and behavioral patterns, suggesting that autonomic dysfunction and anxiety-related features may contribute to the clinical variability observed across the autism spectrum [17].

Heart rate variability (HRV) is a widely used psychophysiological measure of autonomic balance [18] calculated from fluctuations in the time intervals between successive heartbeats [19]. These intervals, typically measured in milliseconds between successive R-peaks on a clean, digitized ECG, form a continuous time series that reflects cardiac-autonomic dynamics [20]. HRV may be potentially useful to identify autonomic profiles for improved diagnostics [21]. Across multiple studies, autistic children exhibit lower cardio-vagal activity as measured by HRV [22]. Low HRV has been linked to social and emotional regulation impairments, making it a candidate biomarker for treatment stratification in autism [23]. Recent findings further support this hypothesis, demonstrating that lower resting-state HRV is associated with greater autism symptom severity and may be modulated by psychotropic medication exposure [24]. Further, research from our team found that the time-domain HRV variable pNN50 predicted the response to propranolol for verbal problem-solving tasks in autism [25], suggesting an a priori target for study. These results reinforce the relevance of HRV as a sensitive physiological marker in both clinical assessment and treatment monitoring in autism.

Furthermore, psychotropic medication is associated with reduced low-frequency HRV, suggesting that such treatments may further contribute to autonomic dysregulation in autism [26]. A meta-analysis examined HRV in autism and found that reduced HRV reactivity during social stress emerged as a compelling physiological biomarker in autism [27]. By demonstrating that autonomic dysregulation was specifically elicited in the context of social interaction, the findings underscore the utility of HRV not only as a static indicator of atypical autonomic function but as a dynamic measure that reflects core socio-communicative challenges in autism. This work thus supports the incorporation of HRV into multimodal assessment frameworks that aim to predict treatment response and tailor interventions to individual autonomic profiles.

Given that some but not all participants experienced reduced anxiety after a 12-week course of propranolol [10], the present study examined HRV as a potential treatment response biomarker to propranolol. Treatment response was evaluated using a clinician-rated assessment of anxiety, and overall effects on core autism symptoms were evaluated through a questionnaire. We hypothesized that autistic children and young adults with the lowest HRV, as measured by pNN50, would have a positive anxiety and behavioral response to propranolol. Group-level (i.e., all participants combined) effects of propranolol on anxiety and behavior and their relationship with other baseline cardiac and time-domain HRV variables were also examined.

## 2. Methods

### 2.1. Participants

Sixty-two individuals (45 males, 17 females), aged 7–24 years (M = 14.8), with a clinical diagnosis of ASD, were enrolled. Inclusion criteria required a formal ASD diagnosis, as confirmed by the Autism Diagnostic Observational Scale (ADOS) [28] or by the Autism Diagnostic Interview-Revised (ADI-R) [29], age between 7 and 24 years, and sufficient cognitive and behavioral capacity to comply with study procedures. The participants were generally regarded as “high functioning” and classified as Level 1—needs support [1]. Additionally, a Full-Scale IQ of 85 on a standardized assessment for individuals 15–24 was required, given the level of social communication tasks that were the primary outcome measures of the parent trial [10]. The ADI-R was selected to assess early developmental symptoms consistent with an autism diagnosis, which is particularly relevant when evaluating adolescents and adults. This tool helps determine whether individuals exhibited core features of autism during childhood—a key component of diagnostic criteria—while the ADOS evaluates the presence of these traits at the time of assessment. This distinction is important, as communication abilities and overall functioning can change substantially with development. ADI-R and ADOS scores were only used to confirm the diagnosis of autism and were not further studied.

Exclusion criteria included the presence of any significant medical condition that could confound autonomic measurements or interfere with propranolol safety. Specifically, individuals were excluded if they had diabetes, thyroid disease, reactive airway disease, bradyarrhythmia, a history of unexplained syncope, or were underweight (defined as less than 20 kg). Participants were also excluded if they had non-autism neurodevelopmental conditions (e.g., dyslexia), other major psychiatric or neurological diagnoses, a history of major head trauma, or a known reaction to adhesives used for sensor placement. In addition, any individual who was pregnant or taking adrenergic agents or other medications known to influence cardiovascular or autonomic function was excluded from participation. All individuals aged 18 and older provided written informed consent. Individuals under 18 provided written assent to the procedures, with consent coming from their parent or legal guardian. All procedures were conducted in accordance with the approval of the Health Sciences Institutional Review Board at the University of Missouri (IRB #2005213). The present study is a secondary analysis of data collected from a previously completed randomized controlled trial of propranolol in autism [30].

Participants completed two structured clinical assessment sessions: one at baseline prior to treatment initiation with propranolol, and a second following either completion of the 12-week double-blind trial or the subsequent open-label propranolol extension. At each assessment time point, resting-state ECG data were collected for HRV analysis, along with clinician-rated CGI-I anxiety scores and parent-reported AIM ratings, as described in detail below.

### 2.2. Procedure

The study employed a double-blind, placebo-controlled randomized clinical trial design followed by a 12-week open-label propranolol extension. During the double-blind phase, participants were randomized to receive either propranolol or a placebo in a parallel-group format. Upon completion of the double-blind phase, all participants were offered the opportunity to transition to an open-label (i.e., unblinded) propranolol phase. Medication dosing was titrated according to body weight to ensure tolerability and safety (weight range: 48–190 lbs.; *M* = 113.6 lbs.). Baseline autonomic function was assessed prior to the initiation of any pharmacologic treatment. Resting-state electrocardiogram (ECG) recordings were obtained in a quiet, temperature-controlled laboratory environment at the University of Missouri Thompson Center for Autism and Neurodevelopment, with participants seated comfortably and instructed to remain awake, relaxed, and still throughout the assessment period. Raw ECG data were acquired using a BIOPAC MP150 Data Acquisition System with an ECG100C amplifier (BIOPAC Systems, Inc., Goleta, CA, USA) configured in a standard three-lead setup (i.e., RA and LA electrodes placed bilaterally under the right and left clavicles respectively, and one LL electrode placed proximally at the lower left rib cage).

Short-term continuous ECG data were recorded for a total of eight minutes, with the initial three minutes designated as an acclimation period and the subsequent five minutes utilized for HRV analysis. ECG traces were removed if they contained significant motion artifacts, as determined by visual inspection by a team member with significant experience in HRV research in autistic people. The ECG data were then processed using a “medium” level of artifact correction using Kubios HRV Scientific software (Version 4.0.0) in alignment with previous research [9,31]. Time- and frequency-domain HRV measures, as well as other relevant cardiac variables (e.g., minimum and maximum heart rate), were calculated, then transferred to a spreadsheet for subsequent statistical analysis.

### 2.3. Measures

#### 2.3.1. Heart Rate Variability (HRV)

Time and frequency domain HRV metrics were derived from baseline ECG recordings using Kubios HRV Scientific software, Version 4.0.0 (Kubios Oy, Kuopio, Finland). Extracted time domain HRV indices included NN50, pNN50, standard deviation of NN intervals (SDNN), standard deviation of heart rate (STD HR), root mean square of successive differences (RMSSD), stress index (i.e., the square root of Baevsky’s stress index as calculated by Kubios HRV software), triangular+ interpolation of the NN interval histogram (TINN), and HRV triangular index [32]. Maximum, minimum, and average heart rate were also calculated.

#### 2.3.2. Treatment Response to Propranolol

Treatment response to propranolol was evaluated using clinician-rated outcome measures at the conclusion of the blinded and open-label phases. Anxiety-related symptom changes were assessed with the Clinical Global Impressions–Improvement (CGI-I) scale [33] that was adapted for anxiety and completed by a blinded clinician. The CGI-I is a widely accepted and validated primary outcome measure in neurodevelopmental and psychiatric clinical trials that is sensitive to meaningful clinical change [34]. The CGI-I captures global shifts in symptom severity relative to baseline using a seven-point scale, where lower scores indicate varying degrees of improvement, a score of 4 reflects no change, and higher scores reflect symptom worsening. For analytic purposes, scores of 1 and 2 were classified as “strong responder,” a score of 3 as “minimal responder,” and scores of 4 or higher as “non-responder.”

Broader behavioral outcomes were assessed using the Autism Impact Measure (AIM), a validated 41-item caregiver-report instrument that evaluates core autism domains, including repetitive behavior, atypical behavior, communication, social reciprocity, and peer interaction [35,36]. The AIM was specifically designed to quantify both the frequency of autism-associated behaviors and the extent to which such behaviors interfere with daily functioning. Importantly, the AIM has demonstrated robust measurement invariance across sex, indicating that it captures core autism symptomatology equivalently in males and females and is not biased toward the more commonly studied male autism phenotype [37]. This validation extends across all five subdomains—repetitive behavior, communication, atypical behavior, social reciprocity, and peer interaction—at the configural, metric, and scalar levels, supporting its psychometric rigor and applicability across diverse presentations of autism. AIM change scores were calculated by subtracting post-treatment ratings from baseline ratings for each participant, providing a dimensional index of behavioral improvement. These change scores were subsequently analyzed in relation to baseline HRV and cardiac metrics using Pearson’s correlation coefficients to examine baseline autonomic predictors of treatment response to propranolol.

### 2.4. Statistical Analysis and Data Reduction

Analyses were performed on data from participants who were randomized and received propranolol in the initial double-blind phase of the study, as well as those who participated in the open-label extension. This allowed maximization of data from all patients receiving propranolol to determine whether HRV predicts response to propranolol. Statistical analyses were performed in JASP Version 0.19.3. All HRV variables were *z*-standardized after the removal of outliers and missing values. After reviewing the HRV data, it was determined that five cases had unusable ECG data; hence, the final sample included 57 cases.

Pearson’s correlation coefficients were then calculated to assess whether HRV and cardiac measures predicted change scores for clinician-rated CGI-I anxiety, AIM total change, and for each AIM subdomain (Repetitive Behavior, Communication, Atypical Behavior, Social Reciprocity, and Peer Interaction). Correlations were calculated for the whole group and for responder classification, coded categorically as non-responder, minimal responder, or strong responder.

## 3. Results

### 3.1. Associations Between Baseline HRV Metrics and CGI-I Anxiety

**Figure 1:**
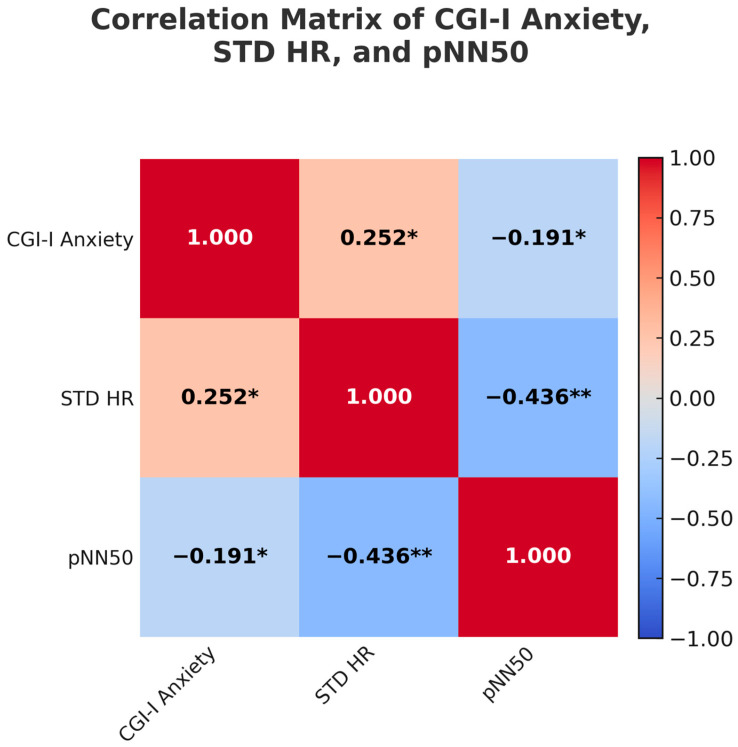
Correlation matrix illustrating relationships among Clinical Global Impression. Improvement (CGI-I) for anxiety, standard deviation of heart rate (STD HR), and percentage of normal RR intervals that differ by 50 milliseconds or more (pNN50). Asterisks indicate statistically significant correlations: * *p* < 0.05, ** *p* < 0.01.

CGI-I Anxiety was positively correlated with STD HR, *r* = 0.25, *p* = 0.047, suggesting that greater variability in heart rate was associated with more improvement in anxiety. CGI-I Anxiety was also negatively correlated with pNN50, *r* = −0.19, *p* = 0.046, indicating that lower parasympathetic tone (as reflected by higher pNN50 values) was associated with greater improvement in anxiety. There were no significant correlations between CGI-I Anxiety and any HRV variables within the responder subgroups. See Figure 1.

### 3.2. Associations Between Baseline HRV and Cardiac Metrics and AIM Change Scores

While our previous work did not show effects of propranolol on non-anxiety outcomes [10], we wished to determine if HRV was associated with behavioral improvement. Pearson correlations were computed to assess whether HRV variables were correlated with AIM total scores as well as with each subdomain of the AIM for the entire sample.

First, mean HR was positively associated with improvements in social reciprocity after treatment with propranolol, *r*(57) = 0.31, *p* = 0.011. Conversely, maximum HR was significantly negatively correlated with social reciprocity, *r*(57) = –0.69, *p* = 0.009, suggesting that greater peak cardiovascular response is linked to less improvement in social functioning after treatment with propranolol.

Next, a small to moderate positive correlation was revealed between AIM change in total scores and RMSSD, *r*(57) = 0.25, *p* = 0.061. This result suggests a trend-level association, with higher RMSSD values being weakly associated with higher AIM change in total scores, though the correlation did not reach statistical significance at the alpha level of 0.05.

Pearson correlation analyses were conducted to examine associations between cardiac metrics and AIM change scores for each behavioral domain as stated in Section 2.4. A small-to-moderate positive correlation was observed between minimum heart rate (Min HR) and AIM Communication change scores, *r*(57) = 0.26, *p* = 0.050, suggesting that higher resting heart rate was associated with improved communication abilities after treatment with propranolol.

With respect to atypical behavior, RMSSD was significantly negatively correlated with atypical behavior, *r*(57) = –0.39, *p* = 0.003, and pNN50 showed a similar pattern, *r*(57) = –0.36, *p* = 0.006. These findings indicate that lower parasympathetic activity is associated with greater improvements in atypical behaviors after treatment with propranolol. Min HR was also positively correlated with improvements in atypical behavior in response to treatment with propranolol, *r*(57) = 0.26, *p* = 0.047.

Together, these findings suggest that both lower parasympathetic tone and reduced cardiac flexibility may be physiological markers for response to treatment for atypical behavior and impaired social reciprocity. See Table 1.

Next, Pearson product–moment correlations were computed between the five AIM behavioral subdomain frequencies and key autonomic indices (Mean HR, Max HR, Mean RR, SDNN, as well as the pNN50) for the 15 participants labeled as “high responders” to propranolol.

Heart-rate variability findings. Improvements in social reciprocity and peer interaction after propranolol were each linked to stronger vagal regulation, indexed by higher SDNN (Social Reciprocity: *r*(13) = –0.58, *p* = 0.022; Peer Interaction: *r*(13) = –0.62, *p* = 0.014). See Table 2.

Heart-rate findings. Improvements in repetitive behavior in response to propranolol were associated with higher baseline mean heart rate, *r*(13) = 0.69, *p* = 0.005, and higher Max HR, *r*(13) = 0.63, *p* = 0.012. Likewise, improved communication after propranolol was correlated positively with Mean HR, *r*(13) = 0.65, *p* = 0.009, and Max HR, *r*(13) = 0.59, *p* = 0.019. Improvements in atypical behavior after propranolol showed a similar pattern (Mean HR: *r*(13) = 0.60, *p* = 0.018; Max HR: *r*(13) = 0.55, *p* = 0.037). In contrast, improved social reciprocity was negatively correlated with Mean HR, *r*(13) = –0.59, *p* = 0.019. See Table 2.

## 4. Discussion

The present study is the first, to our knowledge, to show that baseline autonomic markers forecast the magnitude of clinical response to a 12-week trial of propranolol in autistic children and young adults. Three broad patterns emerged: (a) lower vagal tone (lower pNN50, the hypothesized predictive measure), as well as greater beat-to-beat lability (higher STD HR) predicted larger reductions in clinician-rated anxiety; (b) across the full cohort, indices of sympathetic arousal (higher mean or peak HR) were linked to gains in communication and, along with vagal tone (pNN50) in the reduction in atypical behaviors, whereas higher parasympathetic flexibility (higher RMSSD) tracked improvements in social reciprocity; and (c) among “high responders,” faster baseline HR predicted gains across several behavioral domains, while greater overall HRV (SDNN) predicted social improvement. Below, we situate these findings within the autism, heart-rate variability (HRV), and propranolol literatures, outline mechanistic implications, and note key limitations.

### 4.1. Baseline HRV, Anxiety Relief, and the “Room-to-Improve” Hypothesis

Meta-analytic work shows that autistic individuals generally exhibit blunted vagally mediated HRV at rest, consistent with chronic sympathetic predominance and difficulty down-regulating arousal [27]. In this physiologically “pre-loaded” state, participants who began treatment with lower vagal tone (low pNN50) may have had more capacity to benefit from β-adrenergic blockade, as mirrored by our finding that lower pNN50 predicted superior anxiety improvement. Conversely, higher STD HR before treatment may reflect preserved dynamic range in autonomic output despite low tonic vagal input, enabling more effective dampening of adrenergic surges once β-receptors are blocked. This interpretation aligns with evidence that therapies designed to raise HRV (e.g., HRV-biofeedback) can also alleviate anxiety in autistic samples [38].

Our results also echo HRV work outside autism: in post-traumatic stress disorder, lower resting HRV predicts greater absolute gains from exposure-based treatments [39]. Collectively, these data support a “room-to-improve” model in which sympathetic predominance at baseline signifies greater potential benefit from interventions aimed at reducing adrenergic tone.

### 4.2. Differential Autonomic Signatures of Behavioral Change

Polyvagal theory posits that social approach behaviors are facilitated by a calm physiological state maintained through robust vagal control of the heart [40]. Consistent with this framework, improvements in social reciprocity and peer interaction were associated with greater HRV (higher SDNN) both in the full sample and within high responders, suggesting that effective social engagement may require the capacity to flexibly mobilize the vagal brake.

In contrast, gains in repetitive and atypical behaviors were tied to faster baseline HR and lower HRV—an autonomic profile often observed in autistic individuals with higher externalizing or agitation-driven symptom burdens [27]. One interpretation is that the anxiolytic action of propranolol lowers hyper-arousal, thereby freeing cognitive resources needed for behavioral inhibition. By the same logic, social reciprocity appears to require not merely reduced arousal but active vagal engagement, differentiating it from other behavioral domains.

### 4.3. Mechanistic Alignment with Propranolol Pharmacodynamics

Propranolol is a nonselective β-adrenergic antagonist that crosses the blood–brain barrier and reduces cortical norepinephrine tone. Functional-MRI work in autistic adults from our team showed that an acute dose normalizes atypical long-range connectivity and improves linguistic problem-solving [41]. The current randomized controlled trial—the largest propranolol RCT in autism to date—confirmed benefits for anxiety [10]. Our autonomic findings suggest these behavioral gains arise, at least in part, from rebalancing an overactive sympathetic system: participants with higher sympathetic load (high HR, low pNN50) experienced the greatest symptomatic relief. This mirrors observations in other psychiatric populations where lower baseline HRV predicts more pronounced treatment response [39].

### 4.4. Clinical Implications

Baseline HRV metrics offer a practical tool for personalized medicine because they are noninvasive, inexpensive, and quick to obtain; our findings suggest that these measures could be used to identify autistic individuals who are most likely to benefit from propranolol, especially those exhibiting sympathetic dominance at rest. For participants with chronically low vagal tone who still need greater gains in social engagement, propranolol might be combined with HRV-enhancing interventions—such as paced breathing, biofeedback, or transcutaneous vagal-nerve stimulation—to produce additive benefits. Finally, future propranolol trials that deliberately recruit or stratify participants by autonomic phenotype could reduce sample heterogeneity and increase statistical power, thereby clarifying the behavioral efficacy of the drug.

### 4.5. Limitations and Future Directions

This pilot study relied on a modest sample, limiting power to detect subtle associations within AIM subdomains, and captured anxiety only once via clinician CGI-I ratings. Repeated, ecological assessments delivered through smartphone prompts to participants and caregivers could yield more representative anxiety data while simultaneously recording HRV in real time. Unmeasured factors—sleep, activity level, and daily stress—likely contributed additional variance, and strict exclusion of common comorbidities, restricts generalizability. Longer HRV monitoring windows would further stabilize time-domain estimates. Findings from the secondary HRV measures should be interpreted with caution. Future studies powered to more rigorously determine the salience of these predictive measures are needed, in addition to determining the salience of the relatively modest correlations in this pilot sample.

Larger, multisite trials should therefore stratify participants by autonomic phenotype and integrate HRV with complementary biomarkers (e.g., neuroimaging, neurochemical assays, wearables) in line with multimodal recommendations. Wearable devices that unobtrusively log autonomic and social data across everyday contexts could reveal finer-grained treatment effects, and newer HRV derivatives such as heart-rate fragmentation merit evaluation [42,43]. Clarifying whether baseline HRV predicts long-term maintenance of gains—and whether such prediction is specific to noradrenergic pathways—will extend previous links between adrenergic tone and social reciprocity [9] and cognition [25], as well as incorporation of parent-reported behavioral outcomes [35,36].

Interpretation is further complicated by pooling double-blind and open-label data, which introduces expectancy effects and spontaneous symptom change; replication in fully blinded cohorts is essential. Future studies should also track propranolol interactions with concomitant medications, nutrition, and functional level. Despite these caveats, the current findings refine our understanding of autonomic moderators of β-adrenergic therapy and underscore HRV’s promise as a precision-medicine biomarker in autism.

### 4.6. Conclusions

Our findings highlight potential clinically meaningful links between autonomic physiology and treatment response in autism. Specifically, sympathetic predominance and reduced vagal modulation at baseline forecast greater anxiety and behavioral gains following administration of propranolol for 12 weeks, whereas improvements in social reciprocity align with higher vagal flexibility. HRV thus emerges not only as a descriptive biomarker but also as a potential decision-support tool for precision medicine efforts, informing individualized intervention strategies aimed at optimizing emotional well-being and daily functioning in autistic individuals. However, further studies with a larger sample will be needed to determine its potential role in the clinical setting.

## Figures and Tables

**Table 1:** Significant and Trending Pearson Correlations Between Heart Rate Variability, Cardiac Variables and AIM Change Scores Across Behavioral Domains.

AIM Domain	HRV/Cardiac Variable	*r*	*p*
Communication	Min HR	0.26 ^†^	0.050
Atypical Behavior	RMSSD	–.039 **	0.003
Atypical Behavior	pNN50	–0.36 **	0.006
Atypical Behavior	Min HR	0.26 *	0.047
Social Reciprocity	Mean HR	0.31 *	0.011
Social Reciprocity	Max HR	–0.69 **	0.009

This table presents the correlation coefficients (*r*) and associated *p*-values for relationships between cardiac and heart rate variability (HRV) metrics and AIM change scores for Communication, Atypical Behavior, and Social Reciprocity. Asterisks denote statistically significant correlations (*p* < 0.05 = *, *p* < 0.01 = **), and ^†^ indicates a trend toward significance (*p* < 0.10).

**Table 2:** Significant and Trending Pearson Correlations Between Behavioral Frequencies and Autonomic Indices for High Responders (N = 15).

Behavioral Variable	HRV/Cardiac Variable	*r*	*p*
Repetitive Behavior	Mean HR	0.69 **	0.005
Repetitive Behavior	Max HR	0.63 *	0.012
Communication	Mean HR	0.65 **	0.009
Communication	Max HR	0.59 *	0.019
Atypical Behavior	Mean HR	0.60 *	0.018
Atypical Behavior	Max HR	0.55 *	0.037
Social Reciprocity	Mean HR	–0.59 *	0.019
Social Reciprocity	SDNN	–0.58 *	0.022
Peer Interaction	SDNN	–0.62 *	0.014
Repetitive Behavior	SDNN	–0.46 ^†^	0.085
Atypical Behavior	SDNN	–0.45 ^†^	0.091
Peer Interaction	Mean HR	0.47 ^†^	0.082
Social Reciprocity	Mean RR	–0.49 ^†^	0.068

Note. HR = heart rate; RR = inter-beat interval; SDNN = standard deviation of NN intervals. ^†^ *p* < 0.10 (trend); * *p* < 0.05; ** *p* < 0.01 (two-tailed). Degrees of freedom for all tests = 13.

## Data Availability

No new data were created in this study, as it is a secondary analysis of data from a previous study. Data from that study are available on request only due to privacy restrictions.
